# Interventions to improve hearing aid use in adult auditory rehabilitation: A protocol for an updated systematic review and meta-analysis

**DOI:** 10.1371/journal.pone.0351505

**Published:** 2026-06-24

**Authors:** Sian Calvert, Emma Elizabeth Broome, Mohamad Amin Pourhoseingholi, Jan W. Schoones, Jun Xia, Magdalena Sereda, Paul Bateman, Samuel MacKeith, Shihui Chen, Helen Henshaw

**Affiliations:** 1 Hearing Sciences, Mental Health and Clinical Neurosciences, School of Medicine, University of Nottingham, Nottingham, United Kingdom; 2 National Institute for Health and Care Research (NIHR) Nottingham Biomedical Research Centre, Nottingham, United Kingdom; 3 Leiden University Medical Center, Directorate of Research & Valorisation, Leiden, The Netherlands; 4 Nottingham Ningbo GRADE Centre, University of Nottingham Ningbo China, Ningbo, China; 5 Department of ENT, Oxford University Hospitals, Oxford, United Kingdom; 6 School of Economics, University of Nottingham Ningbo China, Ningbo, China; 7 The First Affiliated Hospital of Ningbo University, Ningbo, China; LSU Health Shreveport, UNITED STATES OF AMERICA

## Abstract

Acquired hearing loss is common among adults, with hearing aids being the primary clinical management option. While hearing aids can improve communication and quality of life, many individuals do not use them consistently or at all. This article presents a protocol for an updated systematic review and meta-analysis of interventions aimed at improving or promoting long-term hearing aid use among adults with hearing loss who are fitted with at least one hearing aid. The review is guided by the Behaviour Change Wheel (BCW) and at its centre the COM-B model (Capability, Opportunity, Motivation – Behaviour), which suggests that the interaction of these three components influences behaviour. The BCW provides a comprehensive framework to understand how interventions target behaviour change and the mechanisms through which they operate. The review objective is to assess the effectiveness of interventions that aim to improve or promote hearing aid use among adults with acquired hearing loss who have at least one hearing aid. Understanding which intervention strategies are effective, and which behavioural functions they target, can help shape future research.

## Introduction

### Background

Hearing loss is a significant public health problem with substantial economic and societal costs, making it a pressing global health concern [1]. The World Health Organisation estimates that 20% of the global population experiences some level of hearing loss, from mild to profound [2], and in 2019, the total global economic impact of unaddressed hearing loss exceeded $980 billion USD [3]. If adult hearing loss is left untreated, it can lead to difficulties with communication that can result in withdrawal, social isolation, depression, and a reduced quality of life [4]. Hearing loss prevalence increases with age [5], and as populations continue to age, it is projected that by 2030, adult-onset hearing loss will rank as the seventh largest contributor to the global disease burden [6].

Around 430 million people, which is over 5% of the global population, need rehabilitation for hearing loss [2]. Hearing aids are the primary clinical management option for hearing loss [7]. Hearing aids pick up sounds from the environment via a microphone and then send the amplified sounds into the ear through a small speaker. Among adults who acknowledge their hearing loss, approximately 78.9% express a willingness to try hearing aids [8]. However, 3% −24% of people fitted with hearing aids do not use them [9,10] and approximately 30% do not use them regularly [11]. Knoetze et al. (2023) report that a range of factors influence hearing aid uptake, e.g., self-reported hearing difficulty, poorer speech perception, demographic factors, motivation and support [12]. Given that hearing aids can significantly enhance communication, social engagement, and overall quality of life [13], it is essential to understand the factors that support consistent, long-term use. Health behaviour change is often guided by theoretical frameworks that explain factors influencing behaviour, including the Behaviour Change Wheel (BCW), in which the central theory (COM-B) frames behaviour in terms of Capability, Opportunity and Motivation [14].

This review is a refined update of a systematic review and meta-analysis [15] and aims to identify interventions that promote the use of hearing aids in adults with acquired hearing loss. In addition to updating the evidence base, this review will use the BCW to understand which factors may influence changes in hearing aid use behaviour. This will give us a better understanding of which intervention strategies are effective, and which behavioural functions they target, so that future research can be tailored to successfully target hearing aid use. This review will systematically synthesise the evidence on interventions to improve hearing aid use and, where appropriate, quantitatively pool the findings through meta-analysis.

### Description of intervention

This review will consider any healthcare intervention that aims to improve or promote hearing aid use in adults with acquired hearing loss who have been fitted with at least one hearing aid. These interventions aim to promote behaviour change in the patient, either directly or indirectly, resulting in a change in the target behaviour – increased hearing aid use. This could include policies, activities, services, or products designed to promote or support people in acting differently from how they would otherwise act [16].

### How the intervention might work

The COM-B is a model that sits in the middle of the BCW, proposing that for a behaviour to be performed, individuals must first have the capability (e.g., knowledge and skills), the opportunity (e.g., social environment and norms), and be motivated (e.g., intentions and desires) to engage in the behaviour [14]. Interventions in this review will be categorised using the Behaviour Change Wheel (BCW), which includes nine Behaviour Change Intervention (BCI) functions. These functions describe the active components through which interventions may influence behaviour. The BCI functions used in this review are summarised in Table 1.

**Table 1:** Description of different behaviour change interventions (BCI) functions [14].

BCI functions	Description of functions
Education	Enhancing understanding and knowledge through explanations, demonstrations and feedback.
Persuasion	Influencing attitudes toward a behaviour by using messages or visuals to make it more or less appealing
Incentivisation	Increasing motivation for a behaviour by promising a positive outcome or avoiding a negative one.
Coercion	Reducing or discouraging a behaviour by introducing potential negative consequences or withholding positive outcomes.
Training	Developing the necessary skills for a behaviour through repeated practice and guidance.
Restriction	Limiting certain behaviours by implementing rules.
Environmental restructuring	Modifying the environment – physical or social – to encourage or discourage specific behaviours.
Modelling	Demonstrating behaviours as examples for other people to replicate.
Enablement	Offering support to help change behaviour in ways not covered by other functions.

Other factors that could influence the effectiveness of BCIs include the *mode of delivery* (i.e., whether the intervention is delivered to individuals in isolation or in a group setting), and *intervention source* (i.e., whether the intervention is self-administered or facilitated by a healthcare professional) [17].

### Why is this review important

Despite evidence that hearing aids improve communication and quality of life, non-use and inconsistent use remain common [18,19]. Interventions to improve hearing aid use are therefore likely to increase these widespread benefits for adults with hearing loss at both the individual and population levels. Hearing loss in midlife is a potentially modifiable risk factor for the development of dementia [20]. Converging evidence suggests that hearing aid use may reduce the rate of cognitive decline for some individuals, while also improving communication, social participation, and quality of life [21]. However, hearing aid non-use remains common, particularly among older adults who may face additional barriers such as reduced such as reduced familiarity with technology and physical limitations (e.g., dexterity issues) [22]. The economic impact of non‐use of hearing aids is significant for both national healthcare providers and individuals who fund their own devices. Finally, should the uptake of hearing aids increase in the future by the introduction of national screening or education programmes [4,23], it is therefore crucial that hearing aid use support is as effective as possible.

This review is important because it builds on earlier research to better understand what could improve and support long-term hearing aid use among adults with hearing loss. By applying the BCW, the review goes beyond just evaluating hearing aid interventions to explore the key behaviour change functions that could influence hearing aid use. This deeper insight can help design more targeted and effective strategies to support long-term hearing aid use, ultimately improving outcomes for adults with hearing loss.

### Objective

To assess the effectiveness of interventions that aim to improve or promote hearing aid use in adults with acquired hearing loss

## Methods

This systematic review has been prospectively registered with PROSPERO (CRD420261295657) and is reported according to the PRSIMA-P checklist (S1 File). The protocol was developed following the recommendations in the Cochrane Handbook for Systematic Reviews of Interventions [24] and the guidelines of the Preferred Reporting Items for Systematic Review and Meta-Analysis (PRISMA-P) [25]. At the time of manuscript submission, study selection is ongoing. Searches, screening, data extraction, and analysis are anticipated to be completed within 12 months of protocol registration (February 2027).

### Step 1. Criteria for selecting studies

#### Participants.

We will include adults (≥ 18 years old) with hearing loss exceeding 20 dB hearing level (HL) in the better ear, which is averaged across the frequencies of 0.5 kHz, 1 kHz, 2 kHz, and 4 kHz, who are fitted with a hearing aid in at least one ear. This definition aligns with the updated criteria from the World Health Organization. It includes individuals with mild (21–40 dB HL), moderate (41–60 dB HL), moderately severe (61–80 dB HL), severe (81–95 dB HL), and profound (>95 dB HL) hearing loss [26].

Where trials do not provide details of participants’ hearing levels, we will assume that those fitted with a hearing aid would have met the criteria for hearing loss. This assumption reflects typical clinical practice, in which hearing aid fitting is generally based on audiometric evidence of hearing loss, and allows inclusion of relevant real-world populations. However, we acknowledge that this may introduce a small risk of misclassification. Where sufficient studies are available, we will explore the impact of this assumption in a sensitivity analysis, excluding studies that do not report participants’ hearing thresholds.

We will include adults with sensorineural, conductive, or mixed hearing loss. Studies involving participants using implantable devices (e.g., cochlear implants) will be excluded. Trials including individuals under 18 years of age will be eligible only if adult-specific data are clearly reported or can be obtained from the study’s authors upon request.

#### Types of interventions.

This review will consider any intervention intended to improve or promote the use of hearing aids by adults who have been fitted with at least one aid. Trials that test or compare hearing aid technology development will be excluded.

This review will focus on interventions that are in addition to the standard hearing aid fitting appointment. Standard care is a one-on-one, in-person hearing aid fitting session, lasting between 45–60 minutes. Typically, hearing aids are fitted by professionals with training in audiology or hearing aid dispensing. A typical hearing aid fitting is expected to include fundamental guidance on the use and maintenance, along with some hands-on practice in its physical management [15].

#### Types of comparisons.

The primary comparison will be intervention vs. standard care. Interventions may also be compared against each other, against no intervention or against ‘standard care’.

Where studies include two or more treatment arms, additional relevant comparisons will be included, where appropriate, following the approach used by Barker et al. [15].

Secondary analyses will examine the relationship between types of BCW intervention function(s) and the impact on hearing aid use behaviour (Table 2).

**Table 2:** Examples of other possible comparison pairs.

Comparisons		
Education Interventions	Versus	Other intervention types or standard care/control.
Persuasion Interventions		
Incentivisation Interventions		
Coercion Interventions		
Training Interventions		
Restriction Interventions		
Environmental Interventions		
Modelling Interventions		
Enablement Interventions		

Intervention components will be characterised using the BCW intervention functions (e.g., Education). As interventions may include multiple functions, BCI functions will be coded as non-mutually exclusive components, with each function recorded as present or absent within each study arm. Where sufficient studies are available, exploratory meta-regression analyses will be conducted to examine whether the presence of individual BCI functions is associated with intervention effectiveness. To account for overlap between intervention components, each function will be coded as a binary moderator in the models. These analyses will be considered exploratory due to the observational nature of component-level comparisons, and causal inferences regarding the effects of individual BCW components will therefore be made with caution.

In addition, subgroup meta-analyses will be conducted comparing interventions that include a given BCI function versus those that do not, where data permit. A component network meta-analysis (CNMA) will be considered only if the number of included studies and the variation in intervention component combinations are sufficient to support reliable estimation; otherwise, this approach will not be undertaken due to risks of model instability and low statistical power.

#### Study types.

We will include studies that have the following design characteristics:

- Randomised controlled trials, including cluster-randomised trials (to avoid the potential for a carry-over phenomenon, cross-over trials will be eligible if the data can be extracted from prior to the crossover).
- Quasi-randomised trials allocate subjects by a non-random process.

There will be no restrictions related to language, year or status of publication.

#### Type of outcome measures.

The review aims to assess interventions that promote hearing aid use after fitting, either by increasing the proportion of those fitted who become hearing aid users or by increasing the amount of hearing aid use per person. While using a hearing aid does not guarantee a benefit, it is an important starting point.

Since hearing loss is a long-term condition and hearing aids are typically a long-term solution, we focus on their use beyond 52 weeks. Follow-up is classified as short-term (≤ 12 weeks), medium-term (> 12 to < 52 weeks), and long-term (> 52 weeks). Short and medium-term follow-up data will be considered indirect evidence if long-term data are available for the same outcome. For the primary analysis, long-term follow-up data will be prioritised. Where multiple follow-up time points are reported within a study, long-term outcomes will be used where available; short- and medium-term outcomes will be included only when long-term data are not reported.

The following outcomes will be analysed in the review, but they will not be used to include or exclude studies.

***Primary outcome measures.*** Hearing aid use. The purpose of this review is to evaluate the extent to which the interventions increased patients’ use of hearing aids. This can be measured in several ways, this review will use the following:

*Adherence*. For this review, we assume that individuals fitted with hearing aids have agreed to this hearing loss management option. We define adherence as the proportion of hearing aids used relative to those fitted. Participants are categorised as either users or non-users. Users are those who wear their hearing aids at least once a week, while non-users are those who do not use them at all or have not worn them in the week leading up to follow-up data collection. If it is unclear how frequently participants use their hearing aids or how they have been categorised as users or non-users, we will reach out to the authors of studies for clarification. If no clarification is provided, the study will be placed in the ‘awaiting assessment’ category.

*Daily hours of use.* Daily hours of hearing aid use will be measured using validated self-report measures that capture average daily usage or through data-logging features built into the hearing aids. Many hearing aids can log when they are switched on, but this may not provide a fully objective measure of actual use. The number of hours a hearing aid is switched on may not accurately reflect the total hours it is worn by the patient. Nevertheless, we intend to use this data as a proxy indicator of usage. Both approaches for gathering data provide continuous data, in terms of hours switched on or the amount of time hearing aids are worn. As this review does not intend to compare these data collection methods, we will integrate self-reported data and data-logging results into our analysis of daily hearing aid use. Where sufficient studies are available, we will explore the potential influence of heterogeneity will be explored through sensitivity or subgroup analyses.

Adverse effects. Adverse effects will include any unintended or undesirable consequences associated with the intervention. These may include, but are not limited to, instances where incorrect advice or inappropriate clinical management could negatively affect patients’ hearing-related outcomes

We will also record patient complaints or concerns, including:

- Unsolved problems related to the physical management of the hearing aid;
- Unsolved problems with symptoms or psychosocial management of hearing loss
- Concerns regarding the intervention, such as the need to make multiple clinic visits.

***Secondary outcome measures.*** For this review, we focus on additional patient-reported outcomes that could be theoretically linked to the use of hearing aids, as proposed by previous reviews [15,27]. The review will not focus on process-related outcomes, for example, quality of care, utilisation, and resource use

We will include validated self-report measures of:

Hearing-specific health-related quality of life (where the main domain is residual/aided participation or activity). When multiple questionnaires are used in a study, these will be ranked in a hierarchy order of importance as follows:

1. Hearing Handicap Inventory for the Elderly (HHIE) [28] or HHI for Adults (HHIA) [29], if the HHIE was not used;
2. Quantified Denver Scale of Communication (QDS) [30];
3. Auditory Disability Preference - Visual Analogue Scale (ADPIVAS) [31];
4. Item 3 or 7 of the International Outcome Inventory for Hearing Aids (IOI-HA) [32]; and
5. any questionnaire not specified above that was relevant to hearing-specific health-related quality of life.

For example, if both the HHIE and QDS are included in a single study, we will use only the HHIE in any meta-analysis.

Health-related quality of life. A proposed ranked hierarchy of self-report outcome measures, ordered as follows:

1. Health Utilities Index Mark 3 (HUI-3) [33];
2. EQ-5D [34];
3. SF-36 [35], or if not reported, other short forms of the SF-36;
4. Glasgow Benefit Inventory (GBI) [36];
5. World Health Organization Disability Assessment Schedule (WHO-DAS) [37];
6. Self-Evaluation of Life Function (SELF) [38]; and
7. any questionnaire not specified above that was relevant to health-related quality of life.

Hearing-aid benefit. A proposed ranked hierarchy of self-report outcome measures, in the following order:

1. Abbreviated Profile of Hearing Aid Benefit (APHAB) [39];
2. Speech, Spatial and Qualities of Hearing (SSQ) [40];
3. Glasgow Hearing Aid Benefit Profile (GHABP, benefit subscale) [41];
4. Item 2 of the International Outcome Inventory for Hearing Aids (IOI-HA) [32]; and
5. any questionnaire not specified above that was relevant to self-report of hearing aid benefit.

Listening ability. A proposed ranked hierarchy of self-report outcome measures, in the following order:

1. Abbreviated Profile of Hearing Aid Benefit (APHAB) [39];
2. Speech, Spatial and Qualities of Hearing (SSQ) [40];
3. Glasgow Hearing Aid Benefit Profile (GHABP, residual disability subscale) [41]; and
4. any questionnaire not specified above that was relevant to self-report of listening ability.

Outcomes will be assessed as short-term (</ = 12 weeks), medium-term (> 12 to < 52), and long-term (> 52 weeks).

### Step 2. The identification of studies: search methods

The Cochrane Ear, Nose and Throat Disorders Group’s Information Specialist designed the search strategy, and author JWS will conduct a systematic search for randomised controlled trials and controlled clinical trials. We will not impose any restrictions on language, publication year, or status. If trial reports are unclear, we may contact the authors for clarification and additional data.

#### Electronic searches.

We will identify published and unpublished studies by searching the subsequent databases from their inception:

- the Cochrane ENT Trials Register (search via the Cochrane Register of Studies to date);
- the Cochrane Central Register of Controlled Trials (CENTRAL) (search via the Cochrane Register of Studies to date);
- Ovid MEDLINE(R) Epub Ahead of Print, In‐Process & Other Non‐Indexed Citations, Ovid MEDLINE(R) Daily and Ovid MEDLINE(R) (1946 to date);
- Ovid Embase (1974 to date);
- EBSCO CINAHL (1982 to date);
- Ovid PsycINFO (1806 to date)
- ClinicalTrials.gov, www.clinicaltrials.gov:
  - search via the Cochrane Register of Studies to date;
  - search via www.clinicaltrials.gov to date;
- World Health Organization (WHO) International Clinical Trials Registry Platform (ICTRP):
  - search via the Cochrane Register of Studies to date;
  - search via https://apps.who.int/trialsearch/ to date).

#### Searching other resources.

If necessary, we will review the reference lists of the identified publications for additional trials and contact the trial authors. Ovid Medline will be searched by the Information Specialist to retrieve relevant systematic reviews, enabling us to review the reference lists for additional trials. We will run non-systematic searches of Google Scholar to identify grey literature and other potential sources of trials. Also, the Information Specialist will search the Web of Science Citation Index for articles that reference Barker (2016) [42] as this review extends the previous review. We will not conduct an additional search for adverse effects; instead, we will consider the adverse effects outlined in the identified studies.

#### Search strategy.

Database-specific subject strategies have been based on the draft search strategies (S2 File); and developed based on an analysis of the search and included studies from previous iterations of a relevant review [15]. Where relevant, we will combine these with subject-specific adaptations of the Cochrane highly sensitive search strategy for identifying randomised controlled trials and controlled clinical trials (as outlined in the Cochrane Handbook for Systematic Reviews of Interventions version 6.5, in the Technical Supplement of Chapter 4) [24].

### Step 3. Data collection and analysis

#### Study selection.

All retrieved records will be independently reviewed by at least two authors to determine whether they are eligible for inclusion in this review. The full-text articles of the retrieved studies will then be reviewed, and the inclusion criteria will be independently applied by each author involved in the study selection process. Disagreements between authors will be discussed, and, if necessary, a third author will be involved until a consensus is reached.

#### Data extraction and management.

Data extraction. At least four authors will independently extract data using an extraction form designed specifically for this review. The data extraction form will be piloted on a small number of articles and revised if needed before formal data extraction begins. Study authors will be contacted for further information if needed or when insufficient data is available in the study.

We will extract the following information: setting, study design, participant characteristics, randomisation and blinding method, power, inclusion and exclusion criteria, intervention (including intervention delivery and source) and control type, pattern of hearing loss, follow-up type and duration of follow-up, attrition or exclusion details, group mean and standard deviation for outcome measures at pre- and post-intervention and follow-up, and results of any statistical tests.

Where more information is needed that is not available in the publication or in an available database, we will contact the authors. If not reported or provided by the authors, we will estimate standard deviations in Review Manager Web [43] using the available data, such as standard errors, confidence intervals, P values and t values. We will make and agree on numeric estimates when data are only available in graphical form.

Data will be independently extracted by two authors; the extracted data will be cross-checked to identify any disagreements, and, if required, a final consensus will be reached by reviewing and discussing the relevant studies. We will extract the data into standard, simple forms that are available upon request from the corresponding author.

We will include ordinal data derived from rating scales only when:

- the measuring instrument has psychometric properties that have been reported in a peer‐reviewed publication; and
- the measuring instrument was not developed or adapted by one of the trialists for that specific trial.

We will primarily use endpoint/change data; if the former is unavailable, we will use change/endpoint data. If required, in the analysis, we will combine endpoints and change data, as mean differences (MDs) are preferred over standardised mean differences (SMDs) throughout.

#### Data management.

Risk of bias. At least two authors will independently assess the risk of bias of the included trials, guided by the Cochrane Handbook for Systematic Reviews of Interventions [24], taking into consideration the following: sequence generation; allocation concealment; blinding; incomplete outcome data; selective outcome reporting; and other sources of bias.

The Cochrane ‘Risk of bias’ tool 2.0 in Review Manager Web [43] will be used to describe the different domains as reported in the trial and then assign a judgement regarding the suitability of each entry: ‘low’, ‘high’, or ‘unclear’ risk of bias.

#### Measure of treatment effect.

For binary outcomes, we will calculate the risk ratio (RR) with 95% confidence intervals (CIs). Continuous outcomes will be summarised as mean differences (MD) with 95% CI. When the same outcome is measured using different scales, standardised mean differences (SMD) (Cohen’s d effect size (ES)) will be used. A positive ES implies that the treatment group had better outcomes than the control group. To make a comparison between trials, we will convert variables that can be reported in different metrics, for example, hours of use (which may be reported as average hours a day, week or month), to the same metric (e.g., average hours a day).

#### Unit of analysis.

The unit of analysis for parallel-group RCTs will be the group mean. Some studies in the review might compare two or more intervention groups or involve clustering. To help prevent unit-of-analysis errors, we will consider alternative analyses for studies with two or more intervention groups and for cluster-randomised trials. For studies with two or more intervention groups, we will consider either combining groups to create a single pairwise comparison or, if this is not appropriate, selecting the pair of interventions most relevant for comparison. For cluster-randomised trials, approximate analyses will be used – effective sample sizes [44]. Cluster-randomised trials will also be adjusted for clustering, using reported or estimated intracluster correlation coefficients (ICCs) to reduce the risk of unit-of-analysis errors.

#### Missing data.

If appropriate and sufficient data from the study are not provided, authors will be contacted to request further details on missing data and the reasons for incomplete data. If there is no response after 14 days, we will impute data ‘missing at random’. If we treat the data as ‘missing not at random’, missing data may affect the overall results; therefore, we will not impute the data. In the latter instance, sensitivity analyses will be conducted with different assumptions and be alerted to possible non-identification or mislabelling of standard errors and standard deviations.

Our imputation methods will follow the recommendations in Cochrane Handbook for Systematic Reviews of Interventions (Chapter 6) [24]. If any data are missing, we will conduct an available case analysis using all reported data for randomised patients available at the end of the study or at the time point of interest, irrespective of the actual treatment received. The quality of outcome assessment will be considered a study limitation (GRADE) rather than a stratifying factor.

#### Assessment of heterogeneity.

We will assess statistical, clinical and methodological heterogeneity. Statistical heterogeneity will be assessed and quantified through visual assessment of the forest plots and by examining the I2 statistic and the Chi2 test in Review Manager Web [43]. Heterogeneity will be considered statistically significant if the P value is less than 0.10 using a Chi2 test with K-1 degrees of freedom. Heterogeneity will be quantified using the I2 statistic, with low, medium, and high ranges of 0% − 40%, 41% − 60%, and 61% − 100%, respectively. Meta‐analyses will be performed using fixed‐effect (in the absence of heterogeneity) and random‐effects modelling (in the presence of heterogeneity). If there is substantial statistical heterogeneity, a subgroup analysis to identify its sources will be conducted.

#### Reporting bias assessment.

Possible publication bias and the impact of individual studies on the overall outcome identified during this review will be investigated. We will attempt to obtain study protocols for the included studies and, if available, examine them to identify any signs of selective outcome reporting. A funnel plot and Egger’s test will be used to judge publication bias if a meta‐analysis includes at least 10 studies.

### Step 4. Data synthesis

If we identify more than one study for a given comparison option and a combination of studies is appropriate, Review Manager Web [40] will be used to perform meta-analyses. A fixed‐effect model will be used to pool data from studies, unless heterogeneity is detected. The RR measure will be used to pool dichotomous data. The SMD measure will be used to pool continuous data, if more than one instrument is used to measure the same outcome.

#### Subgroup analysis and investigation of heterogeneity.

Subgroup analyses will be conducted to explore potential effect modifiers if sufficient data are available. We will restrict this to a very small number of subgroups. If considerable heterogeneity is detected, these subgroup analyses will be used to explore sources of statistical heterogeneity. The planned subgroups are outlined according to:

- Intervention mode of delivery (individual vs. group)
- Intervention source (self-administered vs. facilitated)

#### Sensitivity analysis.

We will perform a sensitivity analysis by removing studies with an elevated risk of bias, to assess the robustness of the meta‐analysis findings. Sensitivity analyses will be used for studies in which data were imputed.

#### Summary of findings and assessment of the certainty of the evidence.

At least two independent authors will apply the GRADE approach, using GRADEpro GDT (https://gradepro.org/) to evaluate the overall certainty of evidence. The evidence certainty will reflect our confidence in the accuracy of the effect estimate and will inform the interpretation of results. The evidence of certainty can be rated as high, moderate, low and very low. A high evidence rating indicates confidence in our estimate of the effect and that more research is unlikely to alter our confidence in the estimate of effect. A very low certainty of evidence indicates that any estimate of the effect acquired is very uncertain.

The GRADE approach rates evidence from RCTs without serious limitations as high certainty. However, several factors can lead to downgrading the evidence to moderate, low or very low. The seriousness of these factors determines the degree of downgrading:

- study limitations (risk of bias);
- inconsistency;
- indirectness of evidence;
- imprecision; and
- dissemination bias.

A ‘Summary of findings’ table, will be included and created in line with the recommendations described in the Cochrane Handbook for Systematic Reviews of Interventions (Chapter 14) [24], for these comparison(s):

- Intervention vs. standard care
- Education interventions versus other intervention types or standard care/control.
- Persuasion interventions versus other intervention types or standard care/control.
- Incentivisation interventions versus other intervention types or standard care/control.
- Coercion interventions versus other intervention types or standard care/control.
- Training interventions versus other intervention types or standard care/control.
- Restriction interventions versus other intervention types or standard care/control.
- Environmental restructuring interventions versus other intervention types or standard care/control.
- Modelling interventions versus other intervention types or standard care/control.
- Enablement interventions versus other intervention types or standard care/control.

In the ‘Summary of findings’ table we will also include the following outcomes:

- Adherence
- Daily hours of use
- Adverse effects
- Hearing-specific health-related quality of life
- Health-related quality of life
- Hearing aid benefit
- Listening ability

## Discussion

This article presents a study protocol for a systematic review and meta-analysis of the literature of interventions to improve hearing aid use in adult auditory rehabilitation. Hearing loss remains a major public health concern, with wide-ranging impacts on communication, social participation, and overall quality of life. Despite hearing aids being the primary clinical intervention for hearing loss, non- or inconsistent use often undermines their potential. This systematic review will synthesise the current evidence on interventions designed to improve hearing aid use in adults with acquired hearing loss. Using the BCW, we will map the behavioural functions of each intervention to better understand which interventions are effective, and the mechanisms through which they may increase hearing aid use.

## Supporting information

S1 File PRISMA-P checklist. (DOCX)

S2 File CENTRAL search strategy. (DOCX)
