# Supplementary material for: Interventions to improve hearing aid use in adult auditory rehabilitation: A protocol for an updated systematic review and meta-analysis
Source: PLoS One. 2026 Jun 24;21(6):e0351505. doi: 10.1371/journal.pone.0351505 (PMC13293450; doi:10.1371/journal.pone.0351505)
Supplement: S2 File — (DOCX) [file pone.0351505.s002.docx]

## Supporting Files

**S1 File. CENTRAL search strategy**

1 MESH DESCRIPTOR Hearing Aids AND CENTRAL:TARGET 285

2 ("hearing aid*"):AB,EH,KW,KY,MC,MH,TI,TO AND CENTRAL:TARGET 881

3 MESH DESCRIPTOR Hearing Loss EXPLODE ALL WITH QUALIFIER RH AND CENTRAL:TARGET 231

4 MESH DESCRIPTOR Correction of Hearing Impairment EXPLODE ALL AND CENTRAL:TARGET 130

5 ((audio* or aural or auditory or "hearing loss") adj3 rehab*):AB,EH,KW,KY,MC,MH,TI,TO AND CENTRAL:TARGET 352

6 #1 OR #2 OR #3 OR #4 OR #5 AND CENTRAL:TARGET 1115

7 MESH DESCRIPTOR Adult AND CENTRAL:TARGET 338782

8 MESH DESCRIPTOR Aged EXPLODE ALL AND CENTRAL:TARGET 213735

9 MESH DESCRIPTOR Presbycusis EXPLODE ALL AND CENTRAL:TARGET 33

10 (older or elder* or aged or aging or "middle age*" or "age related" or acquir* or adult* or Presbycusis or Presbycuses):AB,EH,KW,KY,MC,MH,TI,TO AND CENTRAL:TARGET 892664

11 MESH DESCRIPTOR Middle Aged AND CENTRAL:TARGET 324203

12 #7 OR #8 OR #9 OR #10 OR #11 AND CENTRAL:TARGET 892675

13 #6 AND #12 AND CENTRAL:TARGET 736

14 MESH DESCRIPTOR Presbycusis EXPLODE ALL WITH QUALIFIER RH AND CENTRAL:TARGET 8

15 #13 OR #14 AND CENTRAL:TARGET 736

16 MESH DESCRIPTOR Counseling AND CENTRAL:TARGET 4343

17 MESH DESCRIPTOR Adaptation, Psychological AND CENTRAL:TARGET 4255

18 MESH DESCRIPTOR Orientation AND CENTRAL:TARGET 533

19 MESH DESCRIPTOR Combined Modality Therapy AND CENTRAL:TARGET 15498

20 MESH DESCRIPTOR Treatment Adherence and Compliance EXPLODE ALL AND CENTRAL:TARGET 29245

21 MESH DESCRIPTOR Prosthesis Fitting EXPLODE ALL AND CENTRAL:TARGET 230

22 MESH DESCRIPTOR Patient Education as Topic AND CENTRAL:TARGET 9013

23 MESH DESCRIPTOR Audiology WITH QUALIFIER MT AND CENTRAL:TARGET 4

24 MESH DESCRIPTOR Behavior Therapy AND CENTRAL:TARGET 4702

25 MESH DESCRIPTOR Aftercare AND CENTRAL:TARGET 638

26 ("listening and communication" or LACE):AB,EH,KW,KY,MC,MH,TI,TO AND CENTRAL:TARGET 109

27 (prefitting or Postfitting or "pre-fitting" or "post-fitting" or aftercare or "after care" or "follow-up" or "active fitting"):AB,EH,KW,KY,MC,MH,TI,TO AND CENTRAL:TARGET 244887

28 ("hearing aid*" adj3 fitting):AB,EH,KW,KY,MC,MH,TI,TO AND CENTRAL:TARGET 110

29 ("self manag*" or "management plan*" or "care plan*" or "support tool*" or "fitting protocol" or ghabp or hearing tactic*" or "active fitting" or AO):AB,EH,KW,KY,MC,MH,TI,TO AND CENTRAL:TARGET 12961

30 hearing aid* adj5 ("take up" or "take-up" or "use" or utilis* or utiliz* or "non-use" or adopt* or uptake or reject* or return or orientat* or train* or success*) AND CENTRAL:TARGET 233

31 (counseling or psychosocial):AB,EH,KW,KY,MC,MH,TI,TO AND CENTRAL:TARGET 34802

32 ((patient* or healthcare or "health care" or "hearing aid") adj5 (compliance or cooperat* or co-operat* or adherence or "non-compliance" or noncompliance or "non-adherence" or nonadherence or accept* or nonaccept* or behaviour or behavior or satisfaction or benefit or educat* or promot* or psycholog* or psychosocial or teach* or motivat* or adaptation or perception)):AB,EH,KW,KY,MC,MH,TI,TO AND CENTRAL:TARGET 125440

33 ((listening or communication or audiological or auditory) adj3 (training or skill* or

teaching)):AB,EH,KW,KY,MC,MH,TI,TO AND CENTRAL:TARGET 3345

34 #33 OR #32 OR #31 OR #30 OR #29 OR #28 OR #27 OR #26 OR #25 OR #24 OR #23 OR #22 OR #21 OR #20 OR #19 OR #18 OR #17 OR #16 AND CENTRAL:TARGET 375643

35 #34 AND #15 AND CENTRAL:TARGET 489

36 MESH DESCRIPTOR Hearing Aids EXPLODE ALL WITH QUALIFIER PX AND CENTRAL:TARGET 28

37 #35 OR #36 497
